# The Burden of *Pneumocystis* Pneumonia Infection among HIV Patients in Ethiopia: A Systematic Review

**DOI:** 10.3390/tropicalmed8020114

**Published:** 2023-02-13

**Authors:** Yared Mulu Gelaw, Yonas Deressa Guracho, Florence Robert-Gangneux, Getu Degu Alene, Jean-Pierre Gangneux

**Affiliations:** 1 Department of Health Service and Economics, School of Public Health, College of Medicine and Health Sciences, Bahir Dar University, Bahir Dar H9FX+Q62, Ethiopia; 2 Irset (Institut de Recherche en Santé, Environnement et Travail), UMR_S 1085, Univ Rennes, CHU Rennes, Inserm, EHESP, F-35000 Rennes, France; 3 Faculty of Engineering and Information Sciences, University of Wollongong, Wollongong 2500, Australia

**Keywords:** *Pneumocystis* pneumonia, *Pneumocystis jirovecii*, HIV, systematic review, Ethiopia, Africa, fungal diseases, burden

## Abstract

*Pneumocystis* pneumonia (PCP) is a leading cause of death among patients with AIDS worldwide, but its burden is difficult to estimate in low- and middle-income countries, including Ethiopia. This systematic review aimed to estimate the pooled prevalence of PCP in Ethiopia, the second most densely populated African country. The Preferred Reporting Items for Systematic Reviews and Meta-Analysis (PRISMA) guidelines were used to review published and unpublished studies conducted in Ethiopia. Studies that reported on the prevalence of PCP among HIV-infected patients were searched systematically. Variations between the studies were assessed by using forest plot and I-squared heterogeneity tests. Subgroup and sensitivity analyses were carried out when I2 > 50. The pooled estimate prevalence with 95% CI was computed using a random-effects model of analysis. Thirteen articles, comprising studies of 4847 individuals living with HIV, were included for analysis. The pooled prevalence of PCP was 5.65% (95% CI [3.74–7.56]) with high heterogeneity (I2 = 93.6%, *p* < 0.01). To identify the source of heterogeneity, subgroup analyses were conducted by study design, geographical region, diagnosis methods, and year of publication. PCP prevalence differed significantly when biological diagnostic methods were used (32.25%), in studies published before 2010 (32.51%), in cross-sectional studies (8.08%), and in Addis Ababa (14.05%). PCP prevalence differences of 3.25%, 3.07%, 3.23%, and 2.29% were recorded in studies based on clinical records, published since 2017, follow-up studies, and north-west Ethiopian studies, respectively. The prevalence of PCP is probably underestimated, as the reports were mainly based on clinical records. An expansion of biological diagnostic methods could make it possible to estimate the exact burden of PCP in Ethiopia.

## 1. Introduction

*Pneumocystis* pneumonia (PCP) is a significant opportunistic infection (OI) among patients with human immunodeficiency virus (HIV). It is caused by *Pneumocystis jirovecii*, which remains the major cause of morbidity and mortality among HIV-infected patients, despite the use of highly active antiretroviral therapy [1,2]. Even though the problem is very high in low- and middle-income countries, it has also become a major life-threatening infection among transplant patients and patients with immunosuppressive therapies or cancer in high-income countries [3,4].

PCP was first recognized in undernourished children and in patients who were immune-compromised with malignancies, immunosuppressive therapy, or congenital immune deficiencies, but it was uncommon until 1980. The rate of PCP infection increased with the emergence of HIV [5]. Two decades ago, about 75% of patients with HIV developed PCP during their lifetimes, and it was the cause of two-thirds of AIDS-defining illnesses [6].

The use of highly active antiretroviral therapy and routine primary PCP prophylaxis has diminished the number of patients with HIV-related PCP. In a EuroSIDA study, PCP incidence fell from 4.9 cases per 100 person-years to 0.3 cases per 100 person-years after ART became widely available [7]. Nowadays, in high-resource countries, PCP is mainly diagnosed in newly discovered AIDS cases, or in patients who do not comply with chemoprophylaxis [8].

The incidence of PCP in low- and middle-income countries may differ from that in industrialized ones. In the 1980s, PCP was rare in Africa; the first case in Zambia was detected in 1989, using toluidine blue O stain in a person aged 35 years living with HIV [9]. It was not detected in Uganda in 1989 [10], but in a later study, PCP was diagnosed in 1% of 353 HIV patients admitted to Mulago Hospital who had a cough for more than two weeks, and was responsible for 3% of deaths [11]. In South Africa, it was found in 0.6% of 166 HIV patients in 1992 [12], and it was found in 44% of HIV-infected patients who tested negative on an acid-fast bacilli smear test in 2008 [13]. The problem was found to be very high among children who became infected very shortly after birth, with the prevalence of PCP in these children ranging from 10% to 71% [14,15,16].

Whether the lower incidence of PCP in Africa results from a lack of detection due to poor diagnostic facilities, limited awareness of the disease, or low *Pneumocystis* colonization due to climatic factors is yet to be documented [17]. Indeed, the diagnosis of PCP relies on the microscopic examination of broncho-alveolar fluid after Giemsa staining, but this invasive sampling technique is usually performed only if the first examination of induced sputum did not show tuberculosis (TB), which is the main infection under investigation [11]. In the case of TB diagnosis, other examinations are usually overlooked, and *Pneumocystis* is rarely spotted on the sputum. Molecular diagnosis has become the gold standard for PCP diagnosis in developed countries, but its use remains rare in Africa. In Ethiopia, the first evidence of HIV infection was detected in 1984, and a task force was established in 1985 in response to the HIV epidemic, followed by the national AIDS council in 2000. However, care and treatment were only initiated after the millennium; antiretroviral treatment (ART) for AIDS patients began in 2003, and free ART was launched in 2005 [18,19,20]. OIs were high among people living with HIV in the 2000s; for instance, nearly half (43%) of patients with HIV had developed PCP in 2008 [21].

More recently, the public authority of Ethiopia has been attempting to decrease the infection rate of PCP and other opportunistic infectious diseases among people living with HIV. To achieve this goal, it has adopted the World Health Organization recommendations, i.e., prescribing cotrimoxazole and INH prophylaxis, and providing antiretroviral treatment for all individuals who live with HIV. 

However, studies from different parts of the country have shown that PCP and other OI are still a major public health problem. Moreover, there is no single national study of PCP. Understanding the magnitude of PCP at the national level is of paramount importance to addressing the demand for quality of care, which should lead to reduced morbidity and mortality.

Therefore, this systematic review aimed to synthesize and estimate the prevalence of PCP among HIV-infected people in Ethiopia, the second most densely populated African country, to provide concrete evidence to help policymakers and program managers design appropriate and cost-effective strategies to minimize the burden of PCP.

## 2. Materials and Methods

This systematic review and meta-analysis was conducted to estimate the burden of PCP among HIV-infected people in Ethiopia, in accordance with the Preferred Reporting Items of the Systematic Review and Meta-Analysis (PRISMA) checklist.

### 2.1. Searching Strategy

Different search strategies were used to find studies to be included in the review and meta-analysis. We searched studies published in the English language online in different databases: PubMed, HINARI, Web of Science, Google Scholar, and university repositories. Searches were carried out from 10 December 2020 to 7 January 2021. The search terms used for the PubMed database were (*Pneumocystis* OR pneumonia); OR (*Pneumocystis* OR *Pneumocystis jirovecii* OR *Pneumocystis carinii* OR AIDS-related opportunistic infections); AND (HIV AND (patients OR persons)); AND (Ethiopia). 

Separate search terms also were used to find candidate studies for this study using Google Scholar and Google databases, using the terms “*Pneumocystis*”; OR “Pneumonia, *Pneumocystis*”; OR “*Pneumocystis carinii*”; OR “*Pneumocystis jirovecii*”; OR “AIDS-related opportunistic infections”; AND “HIV”; AND “patients” OR “persons”; AND “Ethiopia”. The selection and exclusion of studies for the systematic review and meta-analysis followed the PRISMA guidelines.

### 2.2. Inclusion and Exclusion Criteria

Epidemiological studies that assessed the prevalence of PCP among HIV/AIDS patients in Ethiopia were included. An article was included if it was conducted among HIV/AIDS patients. Articles published between 1 January 2000 and 1 January 2021 in peer-reviewed journals or university repositories were included. Both cross-sectional and follow-up (trials and cohort) studies published in the English language were included.

### 2.3. Study Selection

All retrieved records were screened against the inclusion criteria. Initially, the decision was based on the title and abstract of the article. For studies that fit the inclusion criteria, or when a definitive decision could not be made based on the title/abstract alone, the full paper was reviewed. The eligibility of each study was assessed independently by two investigators, and the paper was given to a third reviewer to establish a consensus when a discrepancy occurred between the reviewers. 

### 2.4. Data Extraction

Data were collected independently by two individual reviewers from each eligible publication and recorded on a standardized form. During data extraction, variables such as study characteristics, year of publication, design, study population, region, ART coverage, cotrimoxazole preventive therapy (CPT), CD4 count, diagnostic method, and PCP prevalence were captured.

### 2.5. Strategy for Data Synthesis

Data were imported into R studio (R version 4.0.2 (22 June 2020)) from an Excel (Microsoft) sheet for analysis. Both qualitative synthesis and quantitative analysis were performed to present the data extracted from each study. The pooled prevalence of PCP with a 95% confidence interval (95% CI) was calculated using a random-effects model. Heterogeneity between studies was examined using the I squared statistic. According to the test result, an I squared (I^2^) estimate greater than 50% was considered as being indicative of a moderate to high level of heterogeneity [22]. The Dersimonian and Laird random-effects method was used to incorporate an additional between-study component to estimate the variability [23]. Subgroup analyses were performed by outcome ascertainment, study setting, study design, and year of publication as a possible source of heterogeneity between studies. Sensitivity analyses were performed by excluding each study one by one and calculating a pooled prevalence for the remainder of the studies. Publication bias was checked by funnel plot and Egger’s test [24]. Duval and Tweedie’s trim and fill analysis was carried out to adjust the pooled estimate of the prevalence of publication bias [25].

## 3. Results

**Figure 1:**
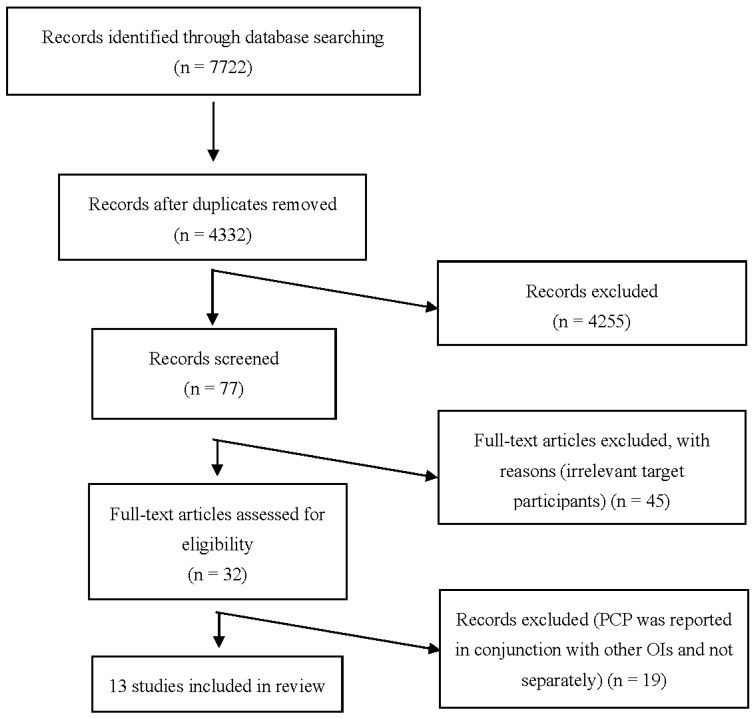
PRISMA flow diagram of identification and selection of studies for the systematic review and meta-analysis. PCP: *Pneumocystis* pneumonia. OIs: opportunistic infections.

Initially, 7722 articles were identified. After screening, 13 articles were included for analysis (Figure 1).

### 3.1. Characteristics of the Studies

This systematic review included thirteen studies published between 2003 and 2020, comprising studies of 4847 individuals living with HIV. The sample size of included studies ranged from 131 [21] to 744 [26]. All except one study [27] included participants ≥15 years. Five studies were carried out in Addis Ababa, Ethiopia’s capital city. Regarding the design, the majority of studies (eight, 61.54%) followed a cross-sectional study design. Among the 13 studies that reported PCP prevalence, 11 (84.6%) used clinical records to assess the diagnosis (Table 1).

### 3.2. Prevalence of PCP

**Figure 2:**
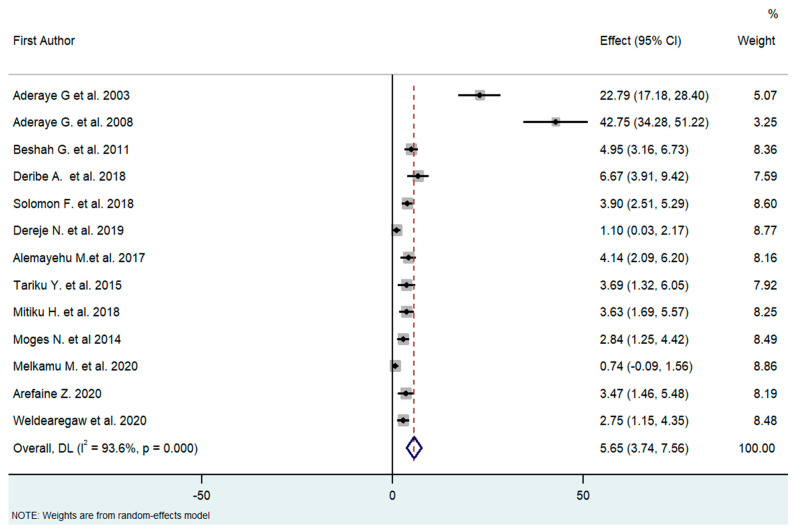
Forest plot of the proportion of PCP among HIV patients in Ethiopia (2003–2020, I^2^ = 93.6%, *p* < 0.01); Q statistics (242.2185, *p* < 0.0001); and test of overall effect (0, z = 5.797, *p* = 0.000). High heterogeneity is suggested in the effect size. To identify the source of heterogeneity, the authors conducted a subgroup analysis [18,21,26,27,28,29,30,31,32,33,34,35,36].

The overall analysis of these 13 studies, using the Dersimonian and Laird random-effects model, found that the pooled prevalence of PCP among people living with HIV was 5.65% (95% CI [3.74–7.56]) with high heterogeneity (I^2^ = 93.6%, *p* < 0.01) (Figure 2).

### 3.3. Subgroup Analysis 

We carried out a subgroup analysis of four variables (PCP ascertainment methods, geographical region where the study was carried out, year of publication, and study design), and a random-effects model was used to test for subgroup differences. 

**Figure 3:**
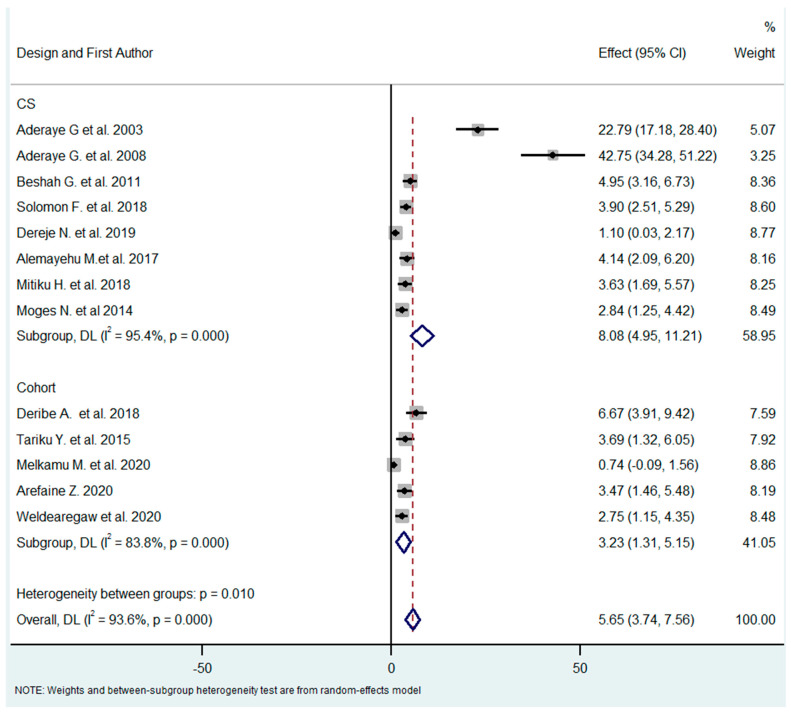
Subgroup analysis by study design [18,21,26,27,28,29,30,31,32,33,34,35,36].

The studies were carried out using different designs (cross-sectional and cohort studies). As PCP is a subacute disease, the design of the study could contribute to a difference in the pooled prevalence related to the timing of the occurrence of the disease. The pooled effect for each subgroup (design) differed substantially (8.08% vs. 3.23% for cross-sectional and cohort/follow-up studies, respectively) in the test of subgroup difference through the random-effects model (I^2^ for CS = 95.4%, I^2^ for cohort = 83.8). This result was not expected since PCP can evolve progressively over several weeks; thus, cross-sectional studies are more likely to miss PCP than follow-up studies (Figure 3).

**Figure 4:**
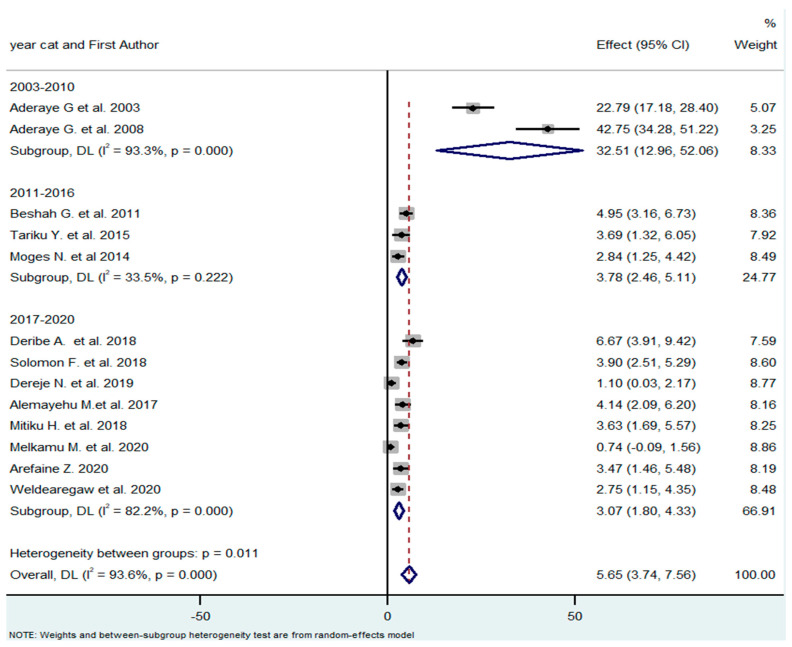
Subgroup analysis by year of publication [18,21,26,27,28,29,30,31,32,33,34,35,36].

The studies were published between 2003 and 2020. As the treatment guidelines were constantly changing within this period, the overall time frame was organized into three subgroups, to take this into account (2003–2010, 2011–2016, and 2017–2020). Since 2017, all individuals living with HIV have been eligible for ART, regardless of their CD4 counts and clinical stages. From 2011 to 2016, people living with HIV began ART when their CD4 count was ≤350 cells/mm^3^, or when they presented with serious HIV clinical infection (WHO clinical stage 3 or 4). Before 2011, people living with HIV began ART when they presented with a CD4 count of ≤ 200 cells/mm^3^, or WHO clinical stage 3 or 4. The pooled effect (prevalence of PCP) of the subgroup differed substantially (32.5% vs. 3.78% vs. 3.07%) (Figure 4).

**Figure 5:**
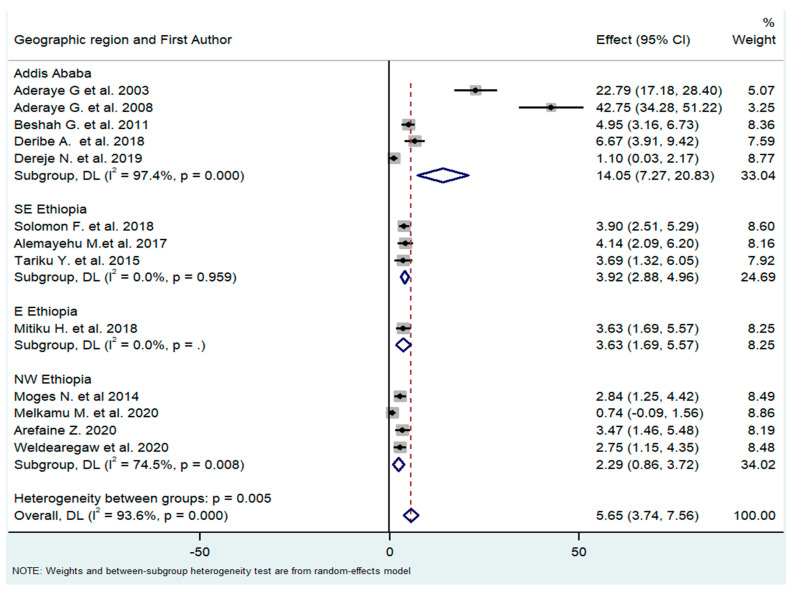
Subgroup analysis by geographical regions where the study was carried out [18,21,26,27,28,29,30,31,32,33,34,35,36].

The studies were carried out in three main regions of the country (Addis Ababa city, eastern Ethiopia, and northern Ethiopia). The pooled effect (prevalence of PCP) of the subgroups differed substantially (14.05%, 3.92%, and 2.29%, respectively). However, the two studies by Aderaye, et al. [21,28] might have outweighed the other studies of the Addis Ababa city subgroup as the heterogeneity of this subgroup was very high (I^2^ = 97%) (Figure 5). 

**Figure 6:**
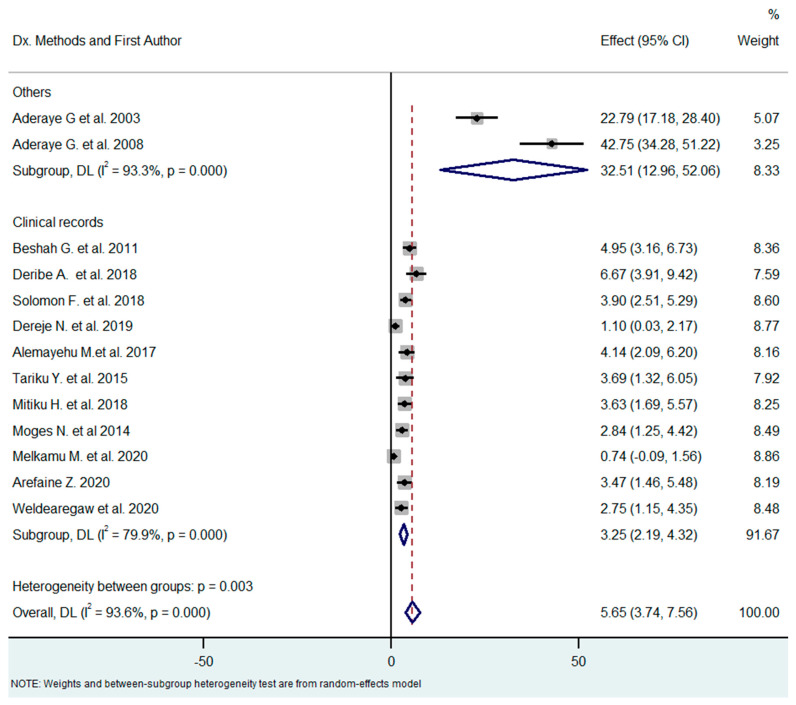
Subgroup analysis by methods of diagnosis [18,21,26,27,28,29,30,31,32,33,34,35,36].

The outcomes (PCP or other pulmonary infection) of the 13 studies were ascertained by different methods (clinical records or biological methods, including nested PCR and direct examination by immunofluorescence). The prevalence of PCP in studies based on clinical charts and in studies based on microbiological diagnosis differed substantially (3.25% vs. 32.51%, respectively), with Q = 24 (*p*-value < 0.0001) meaning that the diagnostic method greatly influenced the estimates of PCP prevalence, with biological diagnosis yielding higher prevalence rates (Figure 6).

**Figure 7:**
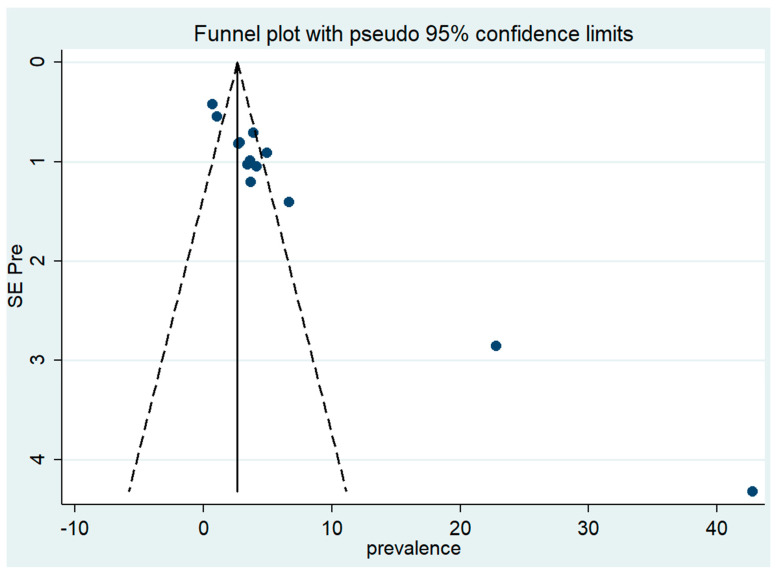
Funnel plot: visual inspection of publication bias for meta-analysis of *Pneumocystis* pneumonia.

One limitation of a systematic review and meta-analysis is that not all studies carried out are published. Additionally, studies that yield statistically significant results are more likely to be submitted and published than works with insignificant results, which can lead to a publication bias that needs to be assessed. This was assessed subjectively through a funnel plot and objectively through Egger’s test. The funnel plot found that, while some studies had statistically significant results, others did not. In addition, there was a trend for large-sized studies to be non-significant to partially significant, while the significance became stronger when the size of the study decreased. The asymmetry was much larger for small studies. This indicates that publication bias might indeed have been present in our analysis (Figure 7).

To measure the publication bias objectively, Egger’s test of the intercept was used to quantify this by performing a statistical test. The results of the test confirmed the presence of a significant funnel with slight asymmetry (Q = 186.248, degree of freedom = 12, *p* < 0.001). Even though this result showed the presence of publication bias, the result of the trim and fill analysis found that data were unchanged after applying trim or if no trim was performed (Q = 186.248, degree of freedom = 12, a moment-based estimate of between-studies variance = 10.544).

## 4. Discussion

Based on modeling using available data from well-defined risk populations, it was estimated that fungi infect approximately 8% of people in Ethiopia every year [37]. According to the authors of that study, the estimated incidence of PCP was 12.1 cases per 100,000 person-years in the whole population. However, as far as the authors were aware, no published systematic review had determined the pooled prevalence of PCP among people who live with HIV in Ethiopia. Therefore, this systematic review and meta-analysis aimed to synthesize and estimate the prevalence of PCP in HIV-infected patients in Ethiopia. Accordingly, the pooled prevalence of PCP in HIV-infected patients in Ethiopia was found to be 5.65% (95% CI [3.74–7.56]).

The prevalence of PCP had shown a decrease over time. It was 33% in 2003–2010, while it dropped to 4% and 3% in 2011–2016 and 2017–2020, respectively (Q = 25.9, *p*-value < 0.0001). A related finding, which is supported by a previous study conducted in Uganda, is that the prevalence of PCP decreases when the CD4 cell count increases [38]. Over the period in question, this links to the expansion of ART coverage, reaching 76% among adults (aged ≥15) in 2017, compared to none in 2003. ART was introduced in 2003 in selected health facilities, and free ART was launched in 2005 [18]. Expanding the accessibility and availability of ART has fundamentally improved the survival rate of people living with HIV by lowering the incidence of OI [39]. Another explanation might be the models for patients’ eligibility to begin ART. Since 2017, Ethiopia has launched a test-and-treat strategy. All HIV-positive patients are eligible for ART, regardless of their WHO clinical stage or CD4 count, and ART initiation is offered on the same day if the patients are mentally prepared to start ART. Before that, from 2011, teenagers and adults only began ART when they presented with a serious or progressed HIV clinical infection (WHO clinical stage 3 or 4), or with a CD4 count ≤ 350 cells/mm^3^. Before 2011, they began ART when they presented with advanced HIV clinical infection (WHO clinical stage 3 or 4) or a CD4 count ≤ 200 cells/mm^3^ [19]. The relatively new test-and-treat system and the commencement of ART before advanced illness may account for the decrease in the incidence of PCP and other OIs in Ethiopia. Today, the rate of patients with a CD4 count < 200 cells/mm^3^ is low among people who start ART and cotrimoxazole preventive therapy (CPT), thus lessening the rate of PCP and other OIs. The hypothesis that the early treatment is responsible for this effect is in agreement with a previous study in America, where ART and CPT diminished the proportion of patients with CD4 counts < 200 cells/mm^3^ [4].

The risk of developing OIs in HIV patients depends on the use of antimicrobial prophylaxis, the level of host resistance, and the virulence of pathogens [40]. Cotrimoxazole (a combination of sulfamethoxazole and trimethoprim) is a broad-spectrum, safe, well-tolerated, low-cost, and widely available antimicrobial agent used as standard care for people living with HIV, and it is utilized in primary healthcare to treat various infections [41]. The Ethiopian national guidelines suggest CPT for individuals living with HIV for the prevention of *Pneumocystis* pneumonia and other OIs, whether bacterial or toxoplasmosis [41]. The guidelines recommend starting CPT when the CD4 count is <350 cell/mm^3^, regardless of WHO clinical stage and/or in WHO clinical stages 3 or 4, regardless of CD4 counts. Recently, the availability and accessibility of CPT reached almost 100% in Ethiopia, which may emphatically impact the decrease in the PCP rate among people living with HIV. Among the studies included in this review, the overall rate of PCP was low; however, the frequency of PCP was high among HIV+ individuals who did not take CPT. Previously, the availability of CPT at health facilities was low, which might explain the high incidence of PCP in the 2000s.

The other reason for the reduction in the PCP rate might be the awareness and testing uptake for HIV, which are increasing in Ethiopia. They make it possible for those living with HIV to start care before they develop complications secondary to HIV. Additionally, adherence to care is increasing due to the reduction of stigma and discrimination in the community.

In addition, the prevalence of PCP varied according to the diagnostic method. It was low in studies that established PCP diagnosis from clinical signs or medical records (3%), compared to studies that used biological diagnostic methods (32%). One possible reason for low PCP prevalence in clinical studies is that PCP might be overlooked if another diagnosis is obtained (for example, TB, using GeneExpert^®^ screening), or it is misdiagnosed as a bacterial infection [42]. As cotrimoxazole is a widely used drug, PCP might be unrecognized, yet successfully treated. Additionally, prevalence rates observed in biological studies could be overestimated if patients were not included prospectively, or if only the most severe patients were screened using biological tools. 

The pooled prevalence was in line with studies carried out in Vietnam (5%) [43] and India (5%) [44]. However, it was lower than those observed in Kenya (37%) [45], Uganda (39%) [46], Botswana (31%) [47], Senegal (43%) [48], Thailand (19%–57%) [49,50], and Chile (38%) [51]. The survey periods might account for this disparity as most of the above studies were carried out before 2010. At that time, the incidence of PCP was high in developing countries, including Ethiopia, as shown by our subgroup analysis. Alternatively, the above-noted discrepancies may be attributed to diagnostic methods, as most of the studies (85%) were based on clinical diagnosis, with the inherent bias discussed above. Our pooled PCP prevalence was higher than in two studies conducted in South Africa (0.5%) [52] and Malawi (1%) [53], but the authors noted that their studies were cohort studies and excluded close to half (48.5%) of participants for various reasons. Therefore, PCP cases could have been missed before they could be followed up. The other reason might be that participants took CPT, which is ideal for both the primary and secondary prophylaxis of PCP [54].

## 5. Conclusions 

This study provides a national figure of PCP in Ethiopia, to help policymakers and program managers design appropriate and cost-effective strategies to minimize the burden of PCP. The prevalence of PCP is decreasing over time. Overall, approximately 6% of individuals infected with HIV experienced PCP, which is relatively low but could be underestimated. The expansion of biological diagnostic methods may enable a greater understanding of the exact burden of PCP in Ethiopia. Molecular diagnosis of PCP is now considered the gold standard [55]. As *Pneumocystis* PCR has been recently added to the list of essential diagnostic tools by the WHO, the question of PCP prevalence is timely, and this review could motivate the implementation of this diagnosis in Ethiopia [56,57].

## Figures and Tables

**Table 1:** Characteristics of the 13 studies included in the systematic review and meta-analysis to estimate the prevalence of *Pneumocystis* pneumonia in Ethiopia among HIV-infected patients.

N°	Region of Study	Year of Study	Age (Year)	Sample Size	Female n (%)	Study Design	PCP Cases n (%)	Diagnosis Method	% of Patients with CD4 ≤ 200/mm^3^ (Except Median and Mean CD4 Count Lines 2 and 3)	% of Patients with HAART	% of Patients with CPT	Reference
1	Addis Ababa	2003	≥15	215	NA	CS	49 (22.7)	Nested PCR	ND	ND	ND	[28]
2	Addis Ababa	2008	>15	131	99 (58)	CS	56 (42.7)	PCR	Mean = 77.9	ND	ND	[21]
3	Addis Ababa	2011	≥18	566	315 (56)	CS	28 (4.9)	Clinical records	ND	100	ND	[29]
4	Addis Ababa	2018	≥20	315	188 (60)	Cohort	21 (6.7)	Clinical records	52.7	100	ND	[30]
5	Addis Ababa	2019	≥18	364	178 (49)	CS	4 (1.1)	Clinical records	77.5	50	ND	[31]
6	SE Ethiopia	2015	≥24	244	105 (43)	Cohort	9 (3.7)	Clinical records	34.0	92.6	100	[32]
7	SE Ethiopia	2017	≥18	362	192 (55)	CS	15 (4.1)	Clinical records	80.4	100	ND	[33]
8	SE Ethiopia	2018	≥18	744	443 (60)	CS	29 (3.9)	Clinical records	48.8	100	ND	[26]
9	E Ethiopia	2015	≥18	358	245 (68)	CS	3 (0.8)	Clinical records	30.7	100	75.7	[34]
10	NW Ethiopia	2014	≥18	423	241 (57)	CS	12 (2.8)	Clinical records	81.3	100	ND	[18]
11	NW Ethiopia	2020	<15	408	223 (55)	Cohort	3 (2.1)	Clinical records	67.9	100	ND	[27]
12	NW Ethiopia	2020	≥16	317	184 (58)	Cohort	7 (2.2)	Clinical records	17	100	99.7	[35]
13	NW Ethiopia	2020	≥18	400	204 (51)	Cohort	11 (2.8)	Clinical records	72.8	100	94.8	[36]
	Total			4847			247 (5.1)					

ND: not determined; CS: cross-sectional; IF: immunofluorescence; CPT: cotrimoxazole preventive therapy.

## Data Availability

No additional data are available.

## References

[1] Center of Diseaese Control (1981). *Pneumocystis* pneumonia Los Angeles. MMWR.

[2] Masur H., Michelis M.A., Greene J.B., Onorato I., Vande Stouwe R.A., Holzman R.S., Wormser G., Brettman L., Lange M., Murray H.W. (1981). An outbreak of community-acquired *Pneumocystis carinii* pneumonia: Initial manifestation of cellular immune dysfunction. N. Engl. J. Med..

[3] Morris A., Lundgren J.D., Masur H., Walzer P.D., Hanson D.L., Frederick T., Huang L., Beard C.B., Kaplan J.E. (2004). Current epidemiology of *Pneumocystis* pneumonia. Emerg. Infect. Dis..

[4] Kaplan J.E., Hanson D., Dworkin M.S., Frederick T., Bertolli J., Lindegren M.L., Holmberg S., Jones J.L. (2000). Epidemiology of human immunodeficiency virus-associated opportunistic infections in the United States in the era of highly active antiretroviral therapy. Clin. Infect. Dis..

[5] Calderón-Sandubete E.J., Varela-Aguilar J.M., Medrano-Ortega F.J., Nieto-Guerrero V., Respaldiza-Salas N., De La Horra-Padilla C., Dei-Cas E. (2002). Historical perspective on *Pneumocystis carinii* infection. Protist.

[6] Hay J.W., Osmond D.H., Jacobson M.A. (1988). Projecting the medical costs of AIDS and ARC in the United States. J. Acquir. Immune Defic. Syndr..

[7] Weverling G.J., Mocroft A., Ledergerber B., Kirk O., Gonzales-Lahoz J., Monforte A.d.A., Proenca R., Phillips A.N., Lundgren J.D., Reiss P. (1999). Discontinuation of *Pneumocystis carinii* pneumonia prophylaxis after start of highly active antiretroviral therapy in HIV-1 infection. Lancet.

[8] Morris A., Norris K.A. (2012). Colonization by *Pneumocystis jirovecii* and its role in disease. Clin. Microbiol. Rev..

[9] Elvin K., Lumbwe C., Luo N., Björkman A., Källenius G., Linder E. (1989). *Pneumocystis carinii* is not a major cause of pneumonia in HIV infected patients in Lusaka, Zambia. Trans. R. Soc. Trop. Med. Hyg..

[10] Serwadda D., Goodgame R., Lucas S., Kocjan G. (1989). Absence of pneumocystosis in Ugandan AIDS patients. AIDS.

[11] Kyeyune R., den Boon S., Cattamanchi A., Davis J.L., Worodria W., Yoo S.D., Huang L. (2010). Causes of early mortality in HIV-infected TB suspects in an East African referral hospital. J. Acquir. Immune Defic. Syndr..

[12] Karstaedt A. (1992). AIDS—The Baragwanath experience. Part III. HIV infection in adults at Baragwanath Hospital. S. Afr. Med. J..

[13] Le Minor O., Germani Y., Chartier L., Lan N.H., Lan N.T., Duc N.H., Laureillard D., Fontanet A., Sar B., Saman M. (2008). Predictors of pneumocystosis or tuberculosis in HIV-infected Asian patients with AFB smear-negative sputum pneumonia. J. Acquir. Immune Defic. Syndr..

[14] Ruffini D.D., Madhi S.A. (2002). The high burden of *Pneumocystis carinii* pneumonia in African HIV-1-infected children hospitalized for severe pneumonia. AIDS.

[15] Zar H.J., Dechaboon A., Hanslo D., Apolles P., Magnus K.G., Hussey G. (2000). *Pneumocystis carinii* pneumonia in South African children infected with human immunodeficiency virus. Pediatr. Infect. Dis. J..

[16] Morrow B.M., Samuel C.M., Zampoli M., Whitelaw A., Zar H.J. (2014). *Pneumocystis* pneumonia in South African children diagnosed by molecular methods. BMC Res. Notes.

[17] Jensen L., Jensen A.V., Praygod G., Kidola J., Faurholt-Jepsen D., Changalucha J., Range N., Friis H., Helweg-Larsen J., Jensen J.S. (2010). Infrequent detection of *Pneumocystis jirovecii* by PCR in oral wash specimens from TB patients with or without HIV and healthy contacts in Tanzania. BMC Infect. Dis..

[18] Moges N., Kassa G. (2014). Prevalence of opportunistic infections and associated factors among HIV positive patients taking anti-retroviral therapy in DebreMarkos Referral Hospital, Northwest Ethiopia. J. AIDS Clin. Res..

[19] Federal Democratic Republic of Ethiopia|Ministry of Health (2014). National Guidelines for Comprehensive HIV Prevention, Care and Treatment.

[20] Seifu L. (2004). Socio-demographic and clinical profile of AIDS patients in Jimma Referral Hospital, Southwest Ethiopia. Ethiop. J. Health Dev..

[21] Aderaye G., Woldeamanuel Y., Asrat D., Lebbad M., Beser J., Worku A., Fernandez V., Lindquist L. (2008). Evaluation of Toluidine Blue O staining for the diagnosis of *Pneumocystis jiroveci* in expectorated sputum sample and bronchoalveolar lavage from HIV-infected patients in a tertiary care referral center in Ethiopia. Infection.

[22] Higgins J.P.T., Green S. (2011). Cochrane Handbook for Systematic Reviews of Interventions Version 5.1.0.

[23] Jackson D., Bowden J., Baker R.D. (2010). How does the DerSimonian and Laird procedure for random effects meta-analysis compare with its more efficient but harder to compute counterparts?. J. Stat. Plan. Inference.

[24] Egger M., Davey Smith G., Schneider M., Minder C. (1997). Bias in meta-analysis detected by a simple, graphical test. BMJ.

[25] Duval S., Tweedie R. (2000). Trim and fill: A simple funnel-plot-based method of testing and adjusting for publication bias in meta-analysis. Biometrics.

[26] Solomon F.B., Angore B.N., Koyra H.C., Tufa E.G., Berheto T.M., Admasu M. (2018). Spectrum of opportunistic infections and associated factors among people living with HIV/AIDS in the era of highly active anti-retroviral treatment in Dawro Zone hospital: A retrospective study. BMC Res. Notes.

[27] Melkamu M.W., Gebeyehu M.T., Afenigus A.D., Hibstie Y.T., Temesgen B., Petrucka P., Alebel A. (2020). Incidence of common opportunistic infections among HIV-infected children on ART at Debre Markos referral hospital, Northwest Ethiopia: A retrospective cohort study. BMC Infect. Dis..

[28] Aderaye G., Bruchfeld J., Olsson M., Lindquist L. (2003). Occurrence of *Pneumocystis carinii* in HIV-positive patients with suspected pulmonary tuberculosis in Ethiopia. AIDS.

[29] Beshah G., Deyessa N. (2001). Study of Prevalence of Opportunistic Infections among HIV/AIDS Patients in Addis Ababa Public Hospitals. Ph.D. Thesis.

[30] Deribe A., Estifanos W. (2018). Magnitude and Determinants of Opportunistic Infections Among HIV/AIDS Patients in Sphmmc, Addis Ababa, Ethiopia, Retrospective Study. JOJ Public Health.

[31] Dereje N., Moges K., Nigatu Y., Holland R. (2019). Prevalence and Predictors of Opportunistic Infections among HIV Positive Adults on Antiretroviral Therapy (On-ART) versus Pre-ART in Addis Ababa, Ethiopia, a Comparative Cross-Sectional Study. HIV/AIDS.

[32] Tariku Y., Yaya Y., Jerene D., Tamiso A. (2015). Incidence of opportunistic infections among adult HIV positive people receiving co-trimoxazole prophylaxis. Int. J. Public Health Sci..

[33] Alemayehu M., Yisehak Y., Alaro W., Alemayehu B. (2017). Opportunistic infections among HIV/AIDS patients taking anti-retroviral therapy at tertiary care hospital in Wolaita zone, Southern Ethiopia. J. AIDS Clin. Res..

[34] Mitiku H., Weldegebreal F., Teklemariam Z. (2015). Magnitude of opportunistic infections and associated factors in HIV-infected adults on antiretroviral therapy in eastern Ethiopia. HIV/AIDS.

[35] Arefaine Z.G., Abebe S., Bekele E., Adem A., Adama Y., Brockmeyer N.H., Coenenberg J., Potthoff A., Gebremeskel T.G. (2020). Incidence and predictors of HIV related opportunistic infections after initiation of highly active antiretroviral therapy at Ayder Referral Hospital, Mekelle, Ethiopia, A retrospective single centered cohort study. PLoS ONE.

[36] Weldearegawi T.Z., Gerensea H., Berihu H., Gidey G., Welearegay M.Z. (2020). The magnitude of opportunistic infections and associated factors in HIV-infected adults on antiretroviral therapy in southern zone Tigray, Ethiopia, a cross-sectional study. Pan Afr. Med. J..

[37] Tufa B.T., Denning D.W. (2019). The burden of fungal infections in Ethiopia. J. Fungi.

[38] Weissberg D., Mubiru F., Kambugu A., Fehr J., Kiragga A., von Braun A., Baumann A., Kaelin M., Sekaggya-Wiltshire C., Kamya M. (2018). Ten years of antiretroviral therapy: Incidences, patterns and risk factors of opportunistic infections in an urban Ugandan cohort. PLoS ONE.

[39] WHO UNAIDS (2005). Progress on Global Access to HIV Antiretroviral Therapy. An Update on ‘3by5’.

[40] Chaisson R.E., Moore R.D. (1997). Prevention of opportunistic infections in the era of improved antiretroviral therapy. J. Acquir. Immune Defic. Synd. Hum. Retrovir..

[41] WHO (2006). Guidelines on Co-Trimoxazole Prophylaxis for HIV-Related Infections among Children, Adolescents and Adults.

[42] Wong M., Back P., Candy G., Nelson G., Murray J. (2006). *Pneumocystis jirovecii* pneumonia in African miners at autopsy. Int. J. Tuberc. Lung Dis..

[43] Louie J.K., Chi N.H., Thao L.T.T., Quang V.M., Campbell J., Chau N.V.V., Rutherford G.W., Farrar J.J., Parry C.M. (2004). Opportunistic infections in hospitalized HIV-infected adults in Ho Chi Minh City, Vietnam: A cross-sectional study. Int. J. STD AIDS.

[44] Rajagopalan N., Suchitra J.B., Shet A., Khan Z.K., Martin-Garcia J., Nonnemacher M.R., Jacobson J.M., Wigdahl B. (2009). Mortality among HIV-infected patients in resource limited settings: A case controlled analysis of inpatients at a community care center. Am. J. Infect. Dis..

[45] Chakaya J., Bii C., Amukoye E., Ouko T., Muita L., Gathua S., Gitau J., Odongo I., Kabanga J., Nagai K. (2003). *Pneumocystis carinii* pneumonia in HIV/AIDS patients at an urban district hospital in Kenya. East Afr. Med. J..

[46] Worodria W., Okot-Nwang M., Yoo S.D., Aisu T. (2003). Causes of lower respiratory infection in HIV-infected Ugandan adults who are sputum AFB smear-negative. Int. J. Tuberc. Lung Dis..

[47] Ansari N.A., Kombe A.H., Kenyon T.A., Mazhani L., Binkin N., Tappero J.W., Gebrekristos T., Nyirenda S., Lucas S.B. (2003). Pathology and causes of death in a series of human immunodeficiency virus-positive and-negative pediatric referral hospital admissions in Botswana. Pediatr. Infect. Dis. J..

[48] Vray M., Germani Y., Chan S., Duc N.H., Sar B., Sarr F.D., Bercion R., Rahalison L., Maynard M., L’Her P. (2008). Clinical features and etiology of pneumonia in acid-fast bacillus sputum smear-negative HIV-infected patients hospitalized in Asia and Africa. AIDS.

[49] Boonsarngsuk V., Sirilak S., Kiatboonsri S. (2009). Acute respiratory failure due to *Pneumocystis* pneumonia: Outcome and prognostic factors. Int. J. Infect. Dis..

[50] Anekthananon T., Ratanasuwan W., Techasathit W., Rongrungruang Y., Suwanagool S. (2004). HIV infection/acquired immunodeficiency syndrome at Siriraj Hospital, 2002: Time for secondary prevention. J. Med. Assoc. Thail..

[51] Chernilo S., Trujillo S., Kahn M., Paredes M., Echevarría G., Sepúlveda C. (2005). Lung diseases among HIV infected patients admitted to the “Instituto Nacional del Torax” in Santiago, Chile. Rev. Med. Chile.

[52] Corbett E.L., Churchyard G.J., Charalambos S., Samb B., Moloi V., Clayton T.C., Grant A.D., Murray J., Hayes R.J., De Cock K.M. (2002). Morbidity and mortality in South African gold miners: Impact of untreated disease due to human immunodeficiency virus. Clin. Infect. Dis..

[53] Van Oosterhout J.J., Laufer M.K., Perez M.A., Graham S.M., Chimbiya N., Thesing P.C., Álvarez-Martinez M.J., Wilson P.E., Chagomerana M., Zijlstra E.E. (2007). *Pneumocystis* pneumonia in HIV-positive adults, Malawi. Emerg. Infect. Dis..

[54] Geresu B., Misganaw D., Beyene Y. (2014). Retrospective evaluation of cotrimoxazole use as preventive therapy in people living with HIV/AIDS in Boru Meda Hospital. BMC Pharmacol. Toxicol..

[55] Guegan H., Robert-Gangneux F. (2019). Molecular diagnosis of *Pneumocystis* pneumonia in immunocompromised patients. Curr. Opin. Infect. Dis..

[56] Bongomin F., Govender N.P., Chakrabarti A., Robert-Gangneux F., Boulware D.R., Zafar A., Oladele R.O., Richardson M.D., Gangneux J.-P., Alastruey-Izquierdo A. (2019). Essential in vitro diagnostics for advanced HIV and serious fungal diseases: International experts’ consensus recommendations. Eur. J. Clin. Microbiol. Infect. Dis..

[57] Orefuwa E., Gangneux J.-P., Denning D.W. (2021). The challenge of access to refined fungal diagnosis: An investment case for low-and middle-income countries. J. Mycol. Med..

